# The Role of Monocytes and Macrophages in Acute and Acute-on-Chronic Liver Failure

**DOI:** 10.3389/fimmu.2018.02948

**Published:** 2018-12-14

**Authors:** Evangelos Triantafyllou, Kevin J. Woollard, Mark J. W. McPhail, Charalambos G. Antoniades, Lucia A. Possamai

**Affiliations:** ^1^Division of Integrative Systems Medicine and Digestive Disease, Imperial College London, London, United Kingdom; ^2^Division of Immunology and Inflammation, Imperial College London, London, United Kingdom; ^3^Department of Inflammation Biology, Institute of Liver Studies, King's College London, London, United Kingdom

**Keywords:** acute liver failure, acute-on-chronic liver failure, monocytes, macrophages, immunosuppression, liver inflammation, damage-associated molecular patterns, pathogen-associated molecular patterns

## Abstract

Acute and acute-on-chronic liver failure (ALF and ACLF), though distinct clinical entities, are considered syndromes of innate immune dysfunction. Patients with ALF and ACLF display evidence of a pro-inflammatory state with local liver inflammation, features of systemic inflammatory response syndrome (SIRS) and vascular endothelial dysfunction that drive progression to multi-organ failure. In an apparent paradox, these patients are concurrently immunosuppressed, exhibiting acquired immune defects that render them highly susceptible to infections. This paradigm of tissue injury succeeded by immunosuppression is seen in other inflammatory conditions such as sepsis, which share poor outcomes and infective complications that account for high morbidity and mortality. Monocyte and macrophage dysfunction are central to disease progression of ALF and ACLF. Activation of liver-resident macrophages (Kupffer cells) by pathogen and damage associated molecular patterns leads to the recruitment of innate effector cells to the injured liver. Early monocyte infiltration may contribute to local tissue destruction during the propagation phase and results in secretion of pro-inflammatory cytokines that drive SIRS. In the hepatic microenvironment, recruited monocytes mature into macrophages following local reprogramming so as to promote resolution responses in a drive to maintain tissue integrity. Intra-hepatic events may affect circulating monocytes through spill over of soluble mediators and exposure to apoptotic cell debris during passage through the liver. Hence, peripheral monocytes show numerous acquired defects in acute liver failure syndromes that impair their anti-microbial programmes and contribute to enhanced susceptibility to sepsis. This review will highlight the cellular and molecular mechanisms by which monocytes and macrophages contribute to the pathophysiology of ALF and ACLF, considering both hepatic inflammation and systemic immunosuppression. We identify areas for further research and potential targets for immune-based therapies to treat these devastating conditions.

## Introduction

The liver is a unique innate immune environment and exerts crucial immune surveillance functions during homeostasis (1, 2). It has a dual blood supply, thus it is constantly exposed to circulating antigens, pathogens, pathogen-associated toxins, and danger signals which reach the liver from the gastrointestinal tract, via the portal vein, or from the systemic circulation via arterial blood (3). Hence, it is an important line of defense and plays a central role in regulating tolerogenic and inflammatory responses (1, 2). For such purposes, the liver shows a high degree of vascularization, slow blood flow through the sinusoidal system and highly permeable fenestrated endothelia allowing direct access to liver immune cells from the blood stream (1, 2). The liver houses an abundant population of tissue-resident macrophages, as intrasinusoidal Kupffer cells (KC), which function as the dominant phagocytes in the liver and compose over 80% of the body's macrophages in states of health. It also contains other myeloid [neutrophils and dendritic cells (DCs)] and lymphoid [T cells, natural killer (NK) cells, and NK T cells] cells that shape innate and adaptive immune responses (2, 4). They are organized in a manner designed to maximize screening for both systemic and gut-derived pathogens, thereby avoiding their systemic spread (5).

While macrophages contribute during the maintenance of homeostasis, they are equally relevant in responses to liver injury, playing key roles in the initiation and progression of liver diseases (1, 2). During injury, the liver macrophage pool is augmented by recruitment of bone-marrow derived monocytes which mature *in situ* into macrophages and contribute to the development and resolution of hepatic inflammation. Macrophage-mediated inflammation may also give rise to systemic consequences. This is perhaps best typified by the conditions of acute liver failure (ALF) and acute-on-chronic liver failure (ACLF), which are characterized by local hepatic inflammation complicated by a systemic inflammation and subsequent systemic immunosuppression. Patients therefore experience symptoms of liver decompensation, accompanied by a systemic inflammatory response that involves endothelial dysfunction and may progress to multi-organ failure along with susceptibility to secondary infections. In this review, we will consider how monocytes and macrophages contribute to the initiation and propagation of local liver inflammation in ALF and ACLF; how they drive systemic immune dysfunction and what immunotherapeutic strategies could be used to target their role.

## The Clinical Syndromes of ALF and ACLF

Acute liver failure (ALF) is a rare condition in which coagulopathy, jaundice, and hepatic encephalopathy arise in the context of an acute hepatic injury and the absence of chronic liver disease (CLD) (6). Various sub-categorizations of ALF have been proposed which use the interval between the development of jaundice and emergence of hepatic encephalopathy to differentiate patients with rapidly progressive “hyperacute” disease from those with a more indolent (“subacute”) clinical course in whom the outcome is generally poorer. In the UK and USA, acetaminophen (paracetamol, APAP) overdose is the commonest cause of ALF and is characterized by the rapid progression of symptoms over a few days (6). In many other parts of the world acute viral hepatitis is the dominant cause of ALF (7). Idiosyncratic drug reactions, hepatic ischemic insults, autoimmune hepatitis, and seronegative disease account for a significant minority of cases and tend to run a slower clinical course (Table 1). A common feature which is shared by all these conditions is hepatocellular loss of a magnitude and at a rate which exceeds the liver's regenerative capacity. Hepatocyte death results in synthetic “loss of function” features such as jaundice and coagulopathy. Significantly, this overwhelming cell death also provokes a robust innate immune response which drives many of the other clinical features of ALF, as will be discussed below.

**Table 1:** Comparison of features of human acute and acute-on-chronic liver failure syndromes.

	**ALF**	**ACLF**
Background liver	Normal	Chronic liver disease ± cirrhosis
Demographics^*^	Mean age: 36 Female preponderance	Mean age: 56 Male preponderance
Causes	Paracetamol/Acetaminophen Other drug-induced liver injury (DILI) Acute viral hepatitis Ischaemia Pregnancy related Autoimmune hepatitis	CLD: Any: Alcohol, chronic viral hepatitis, NASH, other Precipitant: Bacterial infection, alcohol consumption, GI bleed, viral reactivation, *de novo* viral hepatitis, ischaemia, DILI
Clinical features	Coagulopathy, jaundice, hepatic encephalopathy High incidence of SIRS, extrahepatic organ failure and susceptibility to infection	Coagulopathy, jaundice, and extrahepatic organ failure. High incidence of hepatic encephalopathy, SIRS, and susceptibility to infection
Infection susceptibility	Bacterial infection 35–40% Fungal infection 11.2%	37% bacterial infection at diagnosis, increasing to 66% by 4 weeks 2–3.5% fungal infections
Infection onset	Late (>5 days)	Early (< 5 days) and late
Mortality	40% hospital mortality	40–80% hospital mortality
DAMPs/Alarmins	IL-1α, IL-33, ATP, formyl peptides, mitochondrial DNA, cyclophilin A, histones, HMGB1	IL-33, histones, HMGB1

* Patient demographics from large European cohorts, reflecting disease trends in this region. ACLF, acute-on-chronic liver failure; ALF, acute liver failure; ATP, adenosine triphosphate; CLD, chronic liver disease; DAMPs, damage-associated molecular patterns, DILI, drug-induced liver injury; GI, gastrointestinal bleeding; HMGB-1, high-mobility group box-1; IL, interleukin; NASH, non-alcoholic steatohepatitis; SIRS, systemic inflammatory response syndrome

Acute on chronic liver failure (ACLF), in contrast, occurs in patients with pre-existing liver disease. The exact definition of ACLF has been debated in the community, with the Asian Pacific Association for the Study of the Liver (APASL), American Association for the Study of Liver Diseases (AASLD), The European Association for the Study of the Liver-chronic liver failure (EASL-CLIF) consortium and the World Gastroenterology Organization (WGO), all having formalized definitions within the last decade. The WGO working party definition, published in 2014, states: “*ACLF is a syndrome in patients with chronic liver disease with or without previously diagnosed cirrhosis which is characterized by acute hepatic decompensation resulting in liver failure (jaundice* + *prolonged international normalized ratio) and one or more extrahepatic organ failure that is associated with increased mortality within a period of 28 days and up to 3 months from onset*” (8). Key features of this definition are the presence of chronic liver disease and occurrence of acute hepatic dysfunction with synthetic failure that progresses to cause extrahepatic organ failure. The EASL-CLIF definition is important in its distinction that the CLD must be cirrhosis. This more selective population, of patients developing ACLF in the context of cirrhosis, is the best characterized, with much of the evidence base coming from studies that use the EASL-CLIF definition.

Typical events that precipitate ACLF are infections, gastrointestinal bleeds, viral reactivation, and superimposed drug, viral, or ischaemic insults (9, 10) (Table 1). Patients with ACLF experience a high mortality compared to those with uncomplicated decompensation of chronic liver disease and, as would be expected, mortality increases with the severity of extra hepatic organ failure. The 28 day and 90 day mortality from an acute decompensation of cirrhosis are 5 and 14% respectively, whereas for ACLF they range from 22–78 to 41–79% depending on the grade (11).

In both ALF and ACLF, infections are key drivers or life-threatening complicating factors in these syndromes. Discounting the etiological hepatotropic viruses, bacterial infections are the primary microbial clinical issue in liver failure. In ALF bacterial infection occurs in up to a third of patients (12) and is late-onset (>5 days since hospital admission) and predominantly related to gram positive organisms (13). This is postulated to be a consequence of ALF-associated immunosuppression and the invasive nature of critical care support predisposing to nosocomial infections. By contrast, patients with ACLF can suffer from mainly gram negative bacterial infection (14) as a cause for their deterioration, potentiated by high levels of bacterial translocation from the gut, but are also at further risk of secondary nosocomial sepsis during hospital episodes for similar reasons as ALF patients.

## Monocytes and Macrophages in the Initiation of Hepatic Inflammation: Sensing DAMPs and PAMPs

The innate immune system is primed to respond to invading pathogens, through recognition of unique microbial molecular motifs, known as pathogen-associated molecular patterns (PAMPs) (15). PAMPs are recognized via pattern-recognition receptors (PRRs), in a process called structural feature recognition. However, innate immune-mediated inflammation also occurs in the absence of infection. Termed sterile inflammation, this state is induced by release of host-derived products, called damage-associated molecular patterns (DAMPs) during tissue damage (2, 15). DAMPs, which are normally sequestered inside cells, interact with PRRs on immune cells and initiate an inflammatory response (4). In the liver, well described DAMPs include the high-mobility group box-1 (HMGB-1) protein, IL-1α, IL-33, ATP, S100-calcium-binding-protein-A8/A9 (S100A8/9), mitochondrial DNA, histone-associated DNA, purines, heat-shock proteins, and bile acids (2, 3, 15–24). Their activity is mediated through PRRs expressed on liver immune cells such as the toll-like receptors (TLRs), purinergic receptors and the receptor for advanced glycation end-products (RAGE) (2, 15). Ligation of DAMPs to their receptors results in activation of immune cells, which shifts them toward a pro-inflammatory phenotype, thus initiating an inflammatory signal through cytokine and chemokine release, which in turn amplify and sustain the inflammatory response (25).

**Figure 1:**
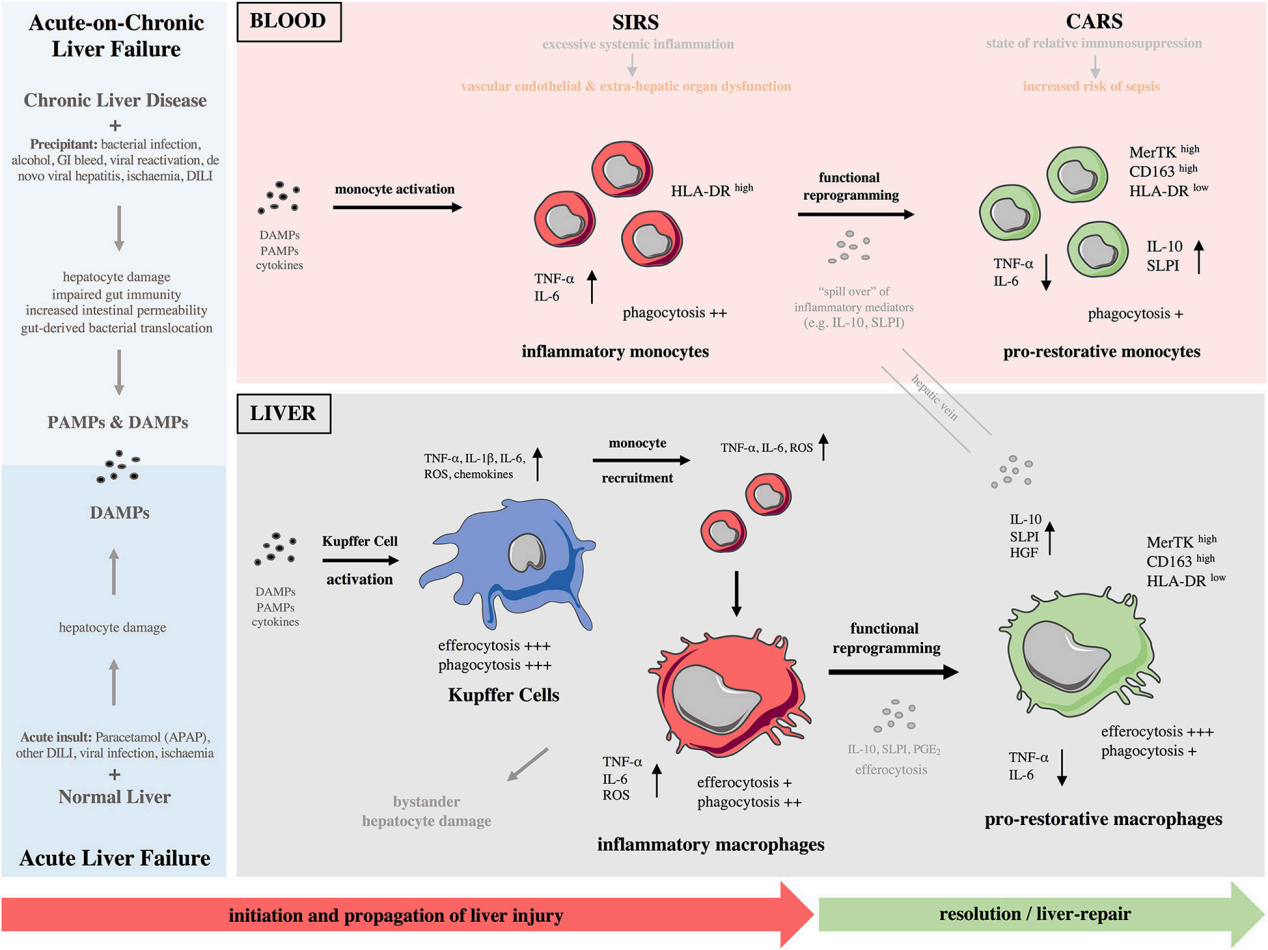
Monocytes and macrophages in the immunopathology of acute and acute-on-chronic liver failure**. (Left)** Different causes lead to development of acute (bottom) and acute-on-chronic (top) liver failure. A major component of the immunopathology of both syndromes is liver inflammation initiated by release of various DAMPs and DAMPs/PAMPs, respectively. **(Right)** During these syndromes, there is a reciprocal interaction of the immune responses between the liver and systemic circulation throughout the different phases. Initiation phase: Kupffer cells become activated after recognition of PAMPs/DAMPs and initiate a pro-inflammatory response. Propagation phase: Bone-marrow derived monocytes are recruited to the liver and differentiate into inflammatory macrophages, expanding the macrophage pool and promoting tissue destruction. During the propagation phase, innate immune activation is self-perpetuating with recruitment of effector cells driving further cytokine and chemokine production; their release to systemic circulation provokes SIRS. These macrophage-derived mediators contribute to vascular endothelial dysfunction and microcirculatory disturbances, resulting in extra-hepatic organ dysfunction. In parallel to SIRS, a CARS develops that is due to release of anti-inflammatory mediators from the liver. Resolution/tissue-repair phase: In response to anti-inflammatory cytokines/mediators and efferocytosis of apoptotic cells, macrophages undergo functional reprogramming toward a pro-restorative phenotype, favoring resolution, and tissue recovery. “Spill over” of anti-inflammatory mediators from the liver to systemic circulation enhances CARS and causes monocyte functional reprogramming toward a pro-restorative phenotype, eventually leading to relative immunosuppression that predisposes susceptibility to infectious complications. CARS, compensatory anti-inflammatory response syndrome; CD, cluster of differentiation; DAMP, damage-associated molecular pattern; DILI, drug-induced liver injury; GI, gastrointestinal bleeding; HGF, hepatocyte growth factor; HLA-DR, human leukocyte antigen-DR; IL, interleukin; MerTK, Mer Tyrosine Kinase receptor; NASH, non-alcoholic steatohepatitis; PAMPs, pathogens-associated molecular patterns; PGE2, prostaglandin E2; ROS, reactive oxygen species; SIRS, systemic inflammatory response syndrome; SLPI, secretory leukocyte protease inhibitor; TNF-α, tumor necrosis factor-alpha.

Liver inflammation can be initiated by various DAMPs and PAMPs and is a major component of the immunopathology of a variety of liver diseases including ALF, ACLF, liver cirrhosis, alcoholic liver disease (ALD), liver fibrosis and cancer (2, 15). As described above, ALF is characterized by massive, rapid hepatocyte death which can occur by either necrotic or apoptotic pathways (26, 27). The result of hepatocyte necrosis is the release of DAMPs (25). Liver-resident KCs highly express various DAMP receptors (e.g., P2X7, TLR4, TLR9 and RAGE) thus mediate the initial response to injury (25). After acetaminophen overdose, oxidative stress and direct mitochondrial damage are induced in hepatocytes, which consequently release DAMPs that can be recognized by KCs (Figure 1). In turn, activated KCs secrete pro-inflammatory cytokines (e.g., TNF-α), reactive oxygen species (ROS), and chemokines (e.g., CCL2) that amplify the pro-inflammatory signal and increase the recruitment of bone-marrow derived cells into the liver, mainly neutrophils and monocytes, thereby enhancing the inflammatory process (2, 15). The sustained KC-released cytokines can also recruit other inflammatory cell subsets, such as eosinophils, DCs, and T cells. While the specific roles of these cell are not fully investigated in ALF, they have been implicated in drug-induced liver injury (2, 15). In ACLF, with a reduced baseline hepatic reserve and longstanding circulatory dysfunction, the initiating event may be lower volume hepatocyte death, which nonetheless causes release of DAMPs and incitement of inflammation (Figure 1). Alternatively, systemic infection may lead to development of ACLF in which inflammation is triggered by an enhanced systemic exposure to PAMPs (10, 28).

In experimental models of ALF and ACLF and in patients with both conditions, a number of DAMPs have been implicated in disease pathogenesis. HMGB-1, perhaps one of the best characterized DAMPs, is a highly conserved chromatin binding protein that is usually located in the cell nucleus (29). During necrotic cell death HMGB-1, along with other nuclear contents, is passively released as cell membrane integrity fails. Released into the local tissue environment and the circulation, HMGB-1 signals through TLR-4 and RAGE receptor on KCs to upregulate NF-κB dependent pro-inflammatory cytokine secretion (20). HMGB-1 in a hyper-acetylated form may also be released by activated monocytes and macrophages. In human paracetamol-induced ALF, HMGB-1 has been shown to be an early biomarker, predicting which patients at presentation will go on to develop acute liver injury (30). High levels of total and acetylated HMGB-1 are also correlated with worse prognosis in patients with ALF (31). Evidence from experimental models suggest HMGB-1 is of mechanistic importance in the pathogenesis of ALF, not merely an epiphenomenon. HMGB-1 neutralizing antibodies are shown to ameliorate injury and reduce bacterial translocation in murine models of paracetamol-induced ALF (21, 32). In patients with hepatitis B related ACLF, HMGB-1 has been shown to be significantly elevated compared with CLD patients, however does not have prognostic value in these patients (33, 34).

Histones are other nuclear structural proteins that when released in an uncontrolled manner during necrotic cell death can act as DAMPs to initiate inflammation. In a study of a cohort of ALF patients predominantly with acute viral hepatitis, extracellular histones were shown to be elevated and correlate with disease severity and outcome (35). In hepatitis B related ACLF levels of extracellular histones were significantly elevated compared with patients with CLD and correlated with clinical evidence of systemic inflammation, severity, and patient outcome (36). Histones act to initiate inflammation through the TLR2 and TLR4 receptors and are shown to activate the nod-like receptor family pyrin-domain containing-3 (NLRP3) inflammasome in immune cells, a signaling pathway leading to IL-1β production. Extracellular histones are directly toxic to endothelial cells and have been linked to the propagation of tissue injury and systemic endothelial dysfunction in sepsis (37). Animal studies and *ex vivo* human work has shown that targeted anti-histone treatments can reduce monocyte pro-inflammatory cytokine production and reduce the severity of ALF (24). Other DAMPs associated with initiation of inflammation in ALF/ACLF include the extracellular ATP signaling via the purinergic P2X7 receptor, extracellular DNA signaling through TLR9, and cyclophilin A (10, 15, 23, 25, 38, 39), as summarized in (Table 1).

PAMPs and DAMPs can collectively contribute to local and systemic inflammation in a variety of liver diseases (25, 40). Several pathologies, including ALF and ACLF, straddle the border between sterile and pathogen-induced inflammation which although are conceptually distinct, largely overlap at a functional level (4). For example, in ALD ethanol-induced liver damage results in released of various DAMPs; however, ethanol exposure also increases intestinal permeability and results in lipopolysaccharide (LPS) leakage, a bacterial-derived PAMP, from commensal intestinal flora to the blood supply to the liver (4, 41). LPS binds to and activates KCs that in turn produce inflammatory cytokines and promote hepatocyte damage (4, 41). In addition, the innate immune system has evolved to use shared PRRs to detect both sterile and infectious insults. Bacterial constituents, such as lipopolysaccharide (LPS), or free bacterial DNA may stimulate the innate immune system via the same PRRs that are used by DAMPs. For instance, TLR4 can be activated by both LPS and HMGB-1 (37, 39). Bacterial infection is one of the commonest precipitating factors in ACLF, and even in the absence of overt sepsis, ACLF may be initiated by events that increase bacterial translocation from the gut into the portal circulation. Taken together, although some forms of sterile liver injury may in fact respond solely to DAMP release, many of these can be complicated by a response to PAMPs.

## Monocytes and Macrophages in the Development of Local Inflammation in Liver Failure Syndromes: Recruitment and Functional Diversity

### Liver Macrophage Plasticity

Macrophages are characterized by their broad diversity and plasticity; in response to injury or infection, they secrete pro-inflammatory cytokines and reactive oxygen/nitrogen species that aid their antimicrobial responses (42, 43). During homeostatic conditions, local micro-environmental cues induce macrophages to adopt phenotypes linked with tissue repair and remodeling (42, 43). Based on their differentiation status, macrophages were traditionally categorized into pro-inflammatory (M1) or anti-inflammatory/wound-healing (M2) (42, 44). However, extensive transcriptomic analyses of human MoMFs, cultured with different stimuli (e.g., cytokines, fatty acids, or lipopolysaccharides), revealed a spectrum of macrophage activation states that are not adequately described by the M1/M2 dichotomy (45). Firstly, the signals received by macrophages in their local microenvironment are diverse and temporally and spatially dynamic (42, 43). Secondly, macrophages not only respond with diverse phenotypes but can also reversibly switch from one type to another (46, 47). A newly proposed way of looking at macrophage polarization is to consider a multidimensional model including the source of macrophages, their specific microenvironments with local signals and a collection of macrophage markers (42, 43).

The liver contains the largest proportion of macrophages among all solid organs in the body (48, 49). Macrophages are a key cellular component of the liver; studies in mouse livers estimate that every 100 hepatocytes are accompanied by 20–40 macrophages (1). Due to their inherent plasticity, liver macrophages flexibly respond to differential environments and adopt to the educative signals arising from parenchymal and other immune cells within the liver (50, 51). Hence, they execute diverse functions during liver inflammation, ranging from tissue-destructive to resolution and pro-restorative roles (47). Liver macrophages secrete high levels of reactive oxygen/nitrogen species and inflammatory cytokines and chemokines, and therefore can regulate both innate and adaptive immune responses. They influence the different phases following liver injury by promoting the clearance of cell debris, extracellular matrix remodeling, tissue regeneration, and inflammatory resolution (2). In line with the multidimensional macrophage model (42, 43), macrophages isolated from injured murine livers express inflammatory (M1-like) and pro-restorative (M2-like) markers simultaneously (52, 53) while can rapidly change their phenotype depending on the local hepatic micro-environmental milieu (54, 55).

### Monocyte Recruitment Into the Liver Following Acute Injury

A prominent feature of acute liver injury is the increased numbers of hepatic macrophages (56–59). Following injury, the macrophage pool of the liver is expanded due to the infiltration of bone marrow derived CCR2+ Ly6C^high^ monocytes which develop into MoMFs (56–59). The CCR2/CCL2 axis is crucial for their recruitment to the liver. Dambach et al. first showed that CCL2 is highly secreted in the mouse liver after APAP overdose while latter work, with several liver injury models applied in CCR2^−/−^ mice, confirmed the importance of CCR2 for monocyte recruitment to the liver (58–61). CCR5 has also been described as crucial for monocyte recruitment in experimental APAP toxicity (62). Human data show that serum and liver tissue CCL2 levels are increased in acetaminophen-induced ALF (AALF) patients who also have increased numbers of S100A8/9+ newly infiltrating monocytes or MoMFs, in necrosis areas within their livers (63). Of note, these patients show increased CCR2 expression in their intermediate, but not classical/non-classical, monocytes (63). The anti-inflammatory liver micro-environment (e.g., CCL2, IL-10, and TGF-β) in human AALF implicates these cells in pro-restorative responses (63). However, the severity of AALF patients correlates inversely with blood monocyte numbers and directly with serum CCL2 levels, suggesting that patients with poorer outcomes recruited more monocytes to the liver (63). In accordance, Mossanen et al. show that CCR2+ cells are increased in the liver of AALF patients and express inflammatory markers, such as S100A9, thus implicating them in propagation of injury (53). Taken together, liver-recruited monocytes play dual roles during liver injury; depending on the disease stage, they can perpetuate inflammation but also promote resolution of inflammation (64).

### *In situ* Development of Liver Monocyte-Derived Macrophages

**Figure 2:**
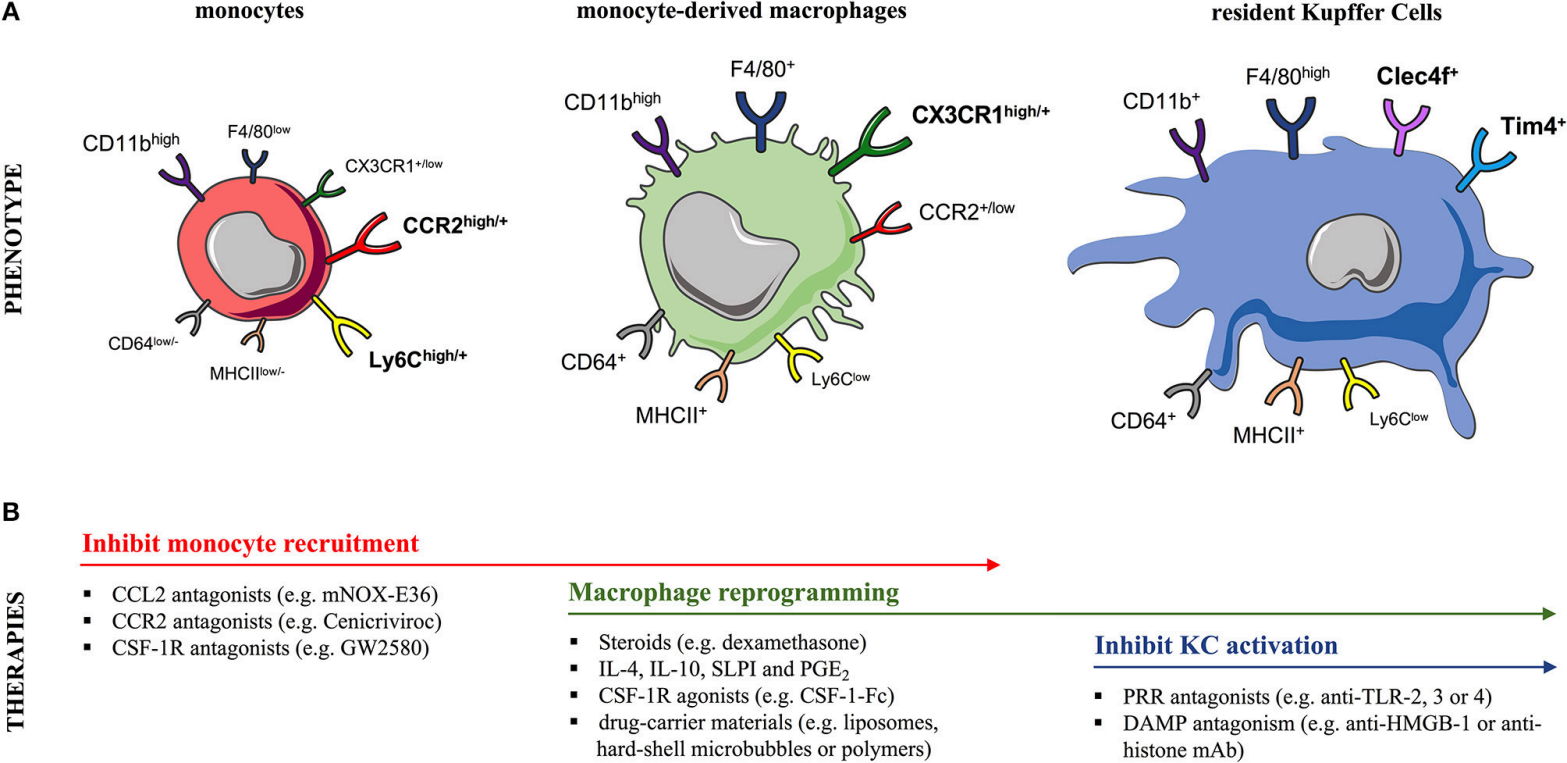
Murine monocyte and liver macrophage subsets and targeted therapeutic strategies**. (A)** In mice, (left) blood and liver-infiltrating monocytes differentially express the markers Ly6C, CCR2, and CX3CR1. During steady-state, the liver macrophage pool can be expanded due to recruitment of circulating (CCR2^+^) Ly6C^high^ monocytes, a process markedly increased after injury. Following their infiltration, monocytes undergo a maturation process into (middle) (CX3CR1^+^) Ly6C_low_ monocyte-derived macrophages (MoMFs) that exhibit a CD11b^high^ F4/80^+^ profile. In contrast, the (right) embryonically derived (CX3CR1^−^) liver-resident Kupffer cells (KCs) are CD11b^+^ F4/80^high^ cells expressing the prototypical markers Clec4F and Tim4. Markers designated in bold are currently used to distinguish these two subsets. **(B)** The table summarizes therapeutic interventions targeting monocyte recruitment, macrophage polarization/differentiation or KC activation in experimental models of acute liver injury. CCL2, CC-chemokine ligand 2; CCR2, CC-chemokine receptor 2; CD, cluster of differentiation; Clec4F, C-type-lectin-domain-family-4-member-F; CSF1R, macrophage colony-stimulating-factor-1 receptor; CX3CR1, CX3C-chemokine receptor 1; DAMP, damage-associated molecular pattern; HMGB-1, high-mobility group box-1; IL, interleukin; MHCII, major histocompatibility complex class II; PRR, pattern-recognition receptor; PGE2, prostaglandin E2; SLPI, secretory leukocyte protease inhibitor; Tim-4, T-cell-immunoglobulin-and-mucin-domain-containing-4; TLR, Toll-like receptor.

Liver MoMFs form a developmentally, phenotypically and functionally distinct subset of macrophages compared to liver-resident KCs (65). Numerous human and mouse studies suggest a potential immune-regulatory role for MoMFs during liver injury. Liver MoMFs are CX3CR1+, derive from blood Ly6C^high^ monocytes while following their infiltration they undergo a maturation process toward Ly6C_low_ MoMFs (52, 65) (Figure 2A). These initially inflammatory CCR2^high^ CX3CR1^low^ monocytes form ring-like structures around injured areas where they differentiate *in situ* into CCR2^low^ CX3CR1^high^ cells, that in turn guide resolution and tissue-repair (8, 29, 66). This maturation process into pro-restorative macrophages is suggested to be driven by the ingestion of cellular debris and augmented by secreted growth factors and cytokines [e.g., macrophage colony-stimulating-factor-1 (CSF1), IL-4, and IL-10] (52, 58, 66, 67). Both resident and infiltrating macrophage populations proliferate in the liver and this expands their numbers during inflammatory conditions (67). Following APAP hepatotoxicity, there is a massive expansion of liver macrophages at 12 h post APAP in mice, that is mainly due to recruitment of MoMFs rather than proliferation of KCs (57, 59, 65). Of note, mouse liver MoMFs are characterized by a gene expression profile that is distinct to circulating Ly6C^high^ monocytes and liver-resident KCs (52, 65). In support of this, increased numbers of newly-infiltrating (S100A8/9+CD68+) macrophages are detected in the liver of AALF patients (53, 63).

In addition to resident KCs, MoMFs apparently contribute to the resolution and tissue-repair processes, as their depletion at different stages of liver injury highlights their critical immune-regulatory roles (68). In both mouse and human ALF, liver-infiltrating MoMFs exhibit a largely anti-inflammatory phenotype and transcriptional signature indicative of a pro-restorative, wound-healing, and regenerative functions (64–66). For instance, mature (CX3CR1^high^ Ly6C^low^) liver MoMFs highly secrete mediators involved in extracellular matrix remodeling, angiogenesis and hepatocyte regeneration (52, 53, 58). This pro-restorative phenotype may be induced by local micro-environmental cues, including CSF1 and secretory leukocyte protease inhibitor (SLPI), that are secreted in the liver of APAP-mice and patients with AALF (64, 66, 67). In keeping with their role in resolving inflammation, two recent studies described that MoMFs regulate neutrophil survival and clearance in APAP liver injury, that is apparently mediated through Mer-Tyrosine-Kinase (MerTK) (66, 69). Recently, Kubes et al. described the infiltration of macrophages from the peritoneal cavity to the liver. Using the thermal and carbon-tetrachloride (CCl_4_)-induced acute liver injury models, they identified a subset of GATA6-expressing peritoneal macrophages as a third population responding to injury (70). These GATA6+ peritoneal macrophages migrate directly through the liver visceral endothelium, and not the vasculature, and aid tissue healing and regeneration processes (70). It remains unclear if this mechanism is restricted to sub-capsular liver lesions in close proximity to the peritoneal cavity. These findings may extend to other models of liver injury, and this new paradigm of avascular macrophage recruitment has opened new avenues for research and potential therapeutic applications (70).

### Kupffer Cell Depletion Following Acute Injury

Mouse liver-resident KCs have an F4/80^high^ CD11b^+^ expression profile in contrast to MoMFs which are F4/80^+^ CD11b^high^ cells (57, 65, 67, 71) (Figure 2A). Owing to their non-monocytic origin, KCs are CX3CR1^neg^ while their expression of other surface markers overlaps with other phagocytes (72, 73). Recent lineage-tracing and transcriptional analyses revealed that KCs express the prototypical markers C-type-lectin-domain-family-4-member-F (Clec4F^+^) and T-cell-immunoglobulin-and-mucin-domain-containing-4 (Tim4^+^) (51, 65, 73, 74) (Figure 2A). The heterogeneity of human liver macrophages is less characterized. There are currently no lineage-specific markers to clearly distinguish human KCs from MoMFs (1, 75). CD68 and CD14 are used as macrophage markers but are commonly expressed by both subsets (1, 53, 76). Most human studies have stained liver tissue of AALF and ACLF patients for CD68 and the pro-inflammatory marker S100A8/9 to discriminate the circulation-derived S100A8/9^+^ MoMFs from resident KCs (53, 63, 64, 66, 77).

Following liver damage in acute liver failure, the absolute number of KCs decreases whereas the liver-recruited monocytes and MoMFs significantly increase as described above (52, 53, 57, 59, 60, 65, 66). In murine models KC depletion occurs at 24–48 h post APAP but full recovery takes place by 120 h, through self-renewal (52, 60, 65). These findings concur with human data showing increased proliferative activity (Ki67+CD68+) of resident macrophages in AALF patients (63). Of note, recent studies revealed that infiltrating bone marrow derived monocytes can replace the KC population, if KCs are completely ablated (54, 74, 78, 79). Following KC depletion in mice, using clodronate-loaded liposomes (CLL), monocytes are able to repopulate the KC and DC populations, giving rise to the full liver myeloid cell heterogeneity within weeks. However, these observations were made in models involving extensive experimental KC depletion (79). In contrast, after KC depletion in APAP injury, KC repopulation relies on self-renewal rather than contribution from monocytes (65). Additional studies have shown that the replacement of KCs by monocytes can result in self-renewing macrophages with similar functional and transcriptional profiles to yolk-sac derived KCs (54, 74). Thus, MoMFs are plastic and are influenced by local signals received within the hepatic microenvironment as they repopulate the KC niche (54, 74).

### Kupffer Cells Are Pivotal for Antimicrobial Defense

The liver is essential for antimicrobial defense and macrophages play a fundamental role in this (3). KCs are highly effective phagocytes that not only recognize, ingest, and degrade cellular debris but also clear foreign material and pathogens (1, 75, 80, 81). Regardless their origin, steady-state KCs are a homogeneous macrophage population with a specific transcriptional program defined by their unique liver niche (54). KCs are exclusively located intravascularly, seeded along the hepatic sinusoidal endothelial cells (HSECs), whereas monocytes and MoMFs can be located extravascularly (58, 70). This optimal location underscores their crucial role in ensuring liver homeostasis by constantly clearing blood-borne pathogens, associated toxins (e.g., LPS) and cellular debris (3). Accordingly, KCs are fully equipped with high expression of Fc (e.g., CD64), scavenger (e.g., CD163), complement, or PRR (e.g., TLR4, TLR9) receptors so they exert antimicrobial responses (1, 75, 82).

Following intravenous injection of *E. coli* bacteria in mice more than 60% of them become trapped in the liver after 10 min, and this proportion can rise to more than 80% in 6 h. Also, intravascular administration of fluorescent-labeled latex microbeads leads to rapid uptake by liver phagocytes with minimal uptake by circulating immune cells or other tissue compartments (e.g., spleen, lung) (67, 79). Mouse experimental studies utilizing flow cytometry and intravital confocal microscopy show that steady-state KCs are the dominant phagocyte in the liver, characterized by enhanced uptake of pH-sensitive *E. coli* bioparticles or GFP-expressing *E. coli*, compared to liver MoMFs and neutrophils (67, 79). However, phagocyte depletion by using CLL in mice revealed that newly-arrived, immature KCs have a reduced phagocytic uptake of *E. coli* for at least 1 month after CCL administration, suggesting a window of hepatic phagocytic dysfunction during the KC maturation process in the liver (79).

KCs can also cooperate with platelets and other non-parenchymal cells (e.g., HSECs or neutrophils) in order to promote pathogen clearance. For example, aggregation of patrolling platelets with KCs facilitates bacterial recognition and clearance during *B. cereus* and *S. aureus* systemic infections in mice, thus conferring protection against sepsis (83). However, recognition and scavenging of bacteria by KCs does not necessarily translate into bacterial clearance. Another recent study has highlighted that KCs can serve as a potential reservoir for *S. aureus*, showing that a small proportion of *S. aureus* overcomes the antimicrobial defense of KCs and replicates within their phagolysosomes, thereby efficiently evading from recruited neutrophils (84). Over time, KCs lyse and release bacteria into the circulation, enabling dissemination to other organs (84). Similarly, KCs were not able to eliminate intracellular bacteria *L. monocytogenes* infection in mice (16). Instead, *L. monocytogenes* induced KC death by necroptosis which triggered the release of hepatocyte-derived danger signals, such as IL-33, and led to monocyte recruitment to the liver; MoMFs ultimately eliminate bacteria and restore homeostasis (16). Work by Sierro et al. recently described an additional liver-resident macrophage population, occupying the hepatic capsule, that is pivotal for the liver antimicrobial defense (73). These liver capsular macrophages (LCMs) are replenished from circulating monocytes at the steady state unlike the embryonically derived KCs. LCMs play a key role in immune surveillance, by sensing peritoneal pathogens and promoting the recruitment of neutrophils toward the capsule to control intrahepatic bacterial dissemination (73).

The depletion of KCs during acute liver injury is likely to have functional and clinical significance in ALF and ACLF. Given that KCs, as the dominant intravascular phagocyte, are responsible for removing live bacterial and microbial products from the circulation and exerting a tolerogenic effect in steady state, their depletion may permit enhanced systemic exposure to DAMPs and persisting bacteraemia. This is an area where further research is required, to define the mechanism of KC depletion and functional significance of the resulting phagocytic defect.

### Liver Macrophages: Drivers of Injury or Promoters of Repair?

The role of liver macrophages in acute liver injury has been controversial. The experimental model of APAP injury in mice is the best explored example, with detailed characterization of macrophage subsets. Initially, KC activation was thought to exacerbate APAP hepatotoxicity in mice (85). However, it is now well-established that KCs are largely depleted after APAP overdose and MoMFs represent the largest macrophage population in the liver (52, 53, 57, 59, 65, 66). The first experiments using gadolinium chloride, a potent KC inactivator, noted protective effects against APAP toxicity, however latter work applying KC depletion prior to APAP demonstrated a beneficial role of KCs (85, 86). Others, by using CLL to deplete most tissue macrophages, showed that macrophage inhibition had a detrimental effect on liver injury (59, 87). Also, mice deficient for NADPH oxidase, an enzyme required for KC oxidative burst, were not protected against APAP injury suggesting that KC-derived oxidant stress is not involved in the injurious APAP process (88).

The role of liver-infiltrating monocytes and their macrophage descendants in acute liver injury has been an area of intense research the last years. Initial studies applying APAP-induced liver injury in CCR2-deficient (CCR2^−/−^) and anti-CCR2 treated mice found increased liver inflammation and delayed tissue regeneration, suggesting that monocytes are crucial for recovery from APAP injury (57, 60). Lack of recruitment of CCR2^high^ CX3CR1^low^ monocytes also results in persistent accumulation of necrotic cells up to 48 h post injury, indicating them crucial for tissue-repair processes (58). Others by using the CCl_4_-induced acute liver injury model found that blood monocyte depletion with CCL resulted in comparable ALT levels at 24 h after CCl_4_ administration, proposing that recruited monocytes do not contribute to the early stages of injury (59). This group further demonstrated that CCR2^−/−^ mice exhibited comparable liver injury at 24 and 48 h following CCl_4_ injection, although liver monocyte numbers were decreased (59). In contrast, others support the idea that monocytes and MoMFs exacerbate liver inflammation. Studies using single-dose CCl_4_ models of acute liver injury and MCP1-deficient (MCP1^−/−^) or CCR2^−/−^ animals suggested that CCL2-recruited CCR2+ monocytes contribute to induction of early injury, showing reduced liver injury at 24 h but comparable levels at 48 h (61, 89). In line with this, two recent studies showed that CCR2+ monocytes infiltrate sites of liver injury as early as 8–12 h following insult (53, 58). Using the APAP experimental model, Mossanen et al. found that CCR2^−/−^ mice had reduced ALT levels and necrosis at 12 h after injury (53). Pharmacological inhibition of monocyte infiltration using a CCR2 antagonist early after APAP dose resulted in reduced ALT levels and necrosis at 12 h following injury, but equivalent injury at 24 and 48 h, supporting the data from the CCl_4_ model (53). Furthermore, this study revealed that the newly-infiltrated (Ly6C^high^) MoMFs exhibit a pro-inflammatory phenotype, as reflected by their cytokine expression (e.g., TNF-α or IL-1β) profile (53). These results suggest that liver monocytes and MoMFs can produce both pro-inflammatory and repair-associated cytokines, however their function clearly depends on the phase (propagation vs. resolution) of liver injury (53, 90). It is important to note the contradictory evidence on the roles of macrophages in ALF. This may be explained by their functional plasticity during the phases (initiation, propagation, and resolution) of liver inflammation (1, 55, 75, 91). These inconsistencies can also be attributed to the hard distinction of macrophage subsets, especially under inflammatory stress, and the different sensitivity of macrophages to the various experimental depletion techniques applied.

## Monocytes and Macrophages and the Systemic Immunopathology of Liver Failure Syndromes

### Systemic Inflammation

#### Development of SIRS and CARS in Liver Failure

The systemic inflammatory response syndrome (SIRS) is the constellation of clinical signs, suggestive of immune activation and the presence of inflammation. It is defined by the presence of any two or more of: fever or hypothermia, tachycardia, tachyopnoea, and white cell response, either leukocytosis, leucopaenia or >10% immature neutrophils (92). SIRS is no longer included in the most recent “Sepsis-3” definition of sepsis, as it was proved to have poor sensitivity and specificity for the identification of patients with infection (93). The lack of specificity is in part because patients with conditions of sterile inflammation like ALF, in which there is a robust innate immune response and high circulating levels of pro-inflammatory cytokines, can exhibit positive SIRS criteria without infection (12). SIRS remains a useful clinical definition for studies investigating immune activation, albeit offering poor discrimination between sterile and sepsis-driven inflammation.

SIRS in ALF is driven by pro-inflammatory cytokine secretion of liver immune cells and is closely associated with the development of extra-hepatic organ dysfunction and adverse outcomes (44). In parallel to the pro-inflammatory response to acute tissue injury, a compensatory anti-inflammatory response syndrome (CARS) develops, as anti-inflammatory cytokines and mediators are concomitantly released from liver macrophages during the initial stages of liver injury (41, 42, 45). CARS is a counter-regulatory, homeostatic mechanism aimed at preventing overwhelming inflammation. Excessive SIRS activation is also hallmark of ACLF. Inflammatory responses in ACLF are imbalanced, ranging from an initial SIRS to subsequent development of a long-lasting CARS (16, 50, 54, 58, 59). ACLF patients are also characterized by high circulating levels of both pro-inflammatory and anti-inflammatory cytokines (22, 50, 63). SIRS and CARS are believed to exert modulatory effects on immune cell effector function in ALF/ACLF, such as monocytes and macrophages, thus contributing to immune dysregulation and defective immune responses to microbial cues (16, 22), as discussed below.

### Systemic Immuneparesis

#### Systemic Immunosuppression in ALF

ALF is a multisystem disorder and immune dysfunction is central to the pathogenesis. Here, the massive hepatic infiltration by myeloid cells is contrasted by immune cell depletion and dysregulation in the systemic circulation (63, 64). From a clinical perspective, it is widely accepted that although the precipitating event of ALF is overwhelming hepatocyte death, mortality occurs due to the profound activation of SIRS and its attendant complications of multi-organ failure, immune dysfunction and recurrent sepsis (12, 94–96). SIRS is driven by pro-inflammatory (e.g., TNF-α, IL-1β, IL-6) cytokine secretion from liver immune cells and is closely associated with the development of extra-hepatic organ dysfunction and adverse outcome in ALF (96). In parallel to the pro-inflammatory response to liver injury, CARS develops as anti-inflammatory cytokines/mediators (e.g., IL-10, SLPI) are concomitantly released from liver macrophages during the initial stages of liver injury (64, 94, 97). Elevated levels of circulating anti-inflammatory cytokines detected in early stages of ALF, such as IL-10, predict poor patient outcome (64, 94, 97). ALF shares striking similarities with systemic sepsis and septic shock with regards to the features of systemic inflammation, multi-organ failure and peripheral immunosuppression, thus may share pathological mechanisms (94, 98). Immunosuppression is considered the predominant driving force for mortality in those patients while accumulating evidence shows that sepsis causes major defects in innate and adaptive immunity, leading to compromised host antimicrobial defense (99, 100). “Septic” monocytes show reduced HLA-DR expression and diminished ability to produce pro-inflammatory cytokines (e.g., TNF-α, IL-6, and IL-12) in response to TLR agonists, findings consistent with endotoxin tolerance (99, 100). In contrast, anti-inflammatory mediator (e.g., IL-10) secretion is unaltered or even augmented (99, 100), and these characteristics may account for the increased patient susceptibility to infections in sepsis (101). Of note, sepsis is one of the most important causes of mortality in ALF patients (12).

**Figure 3:**
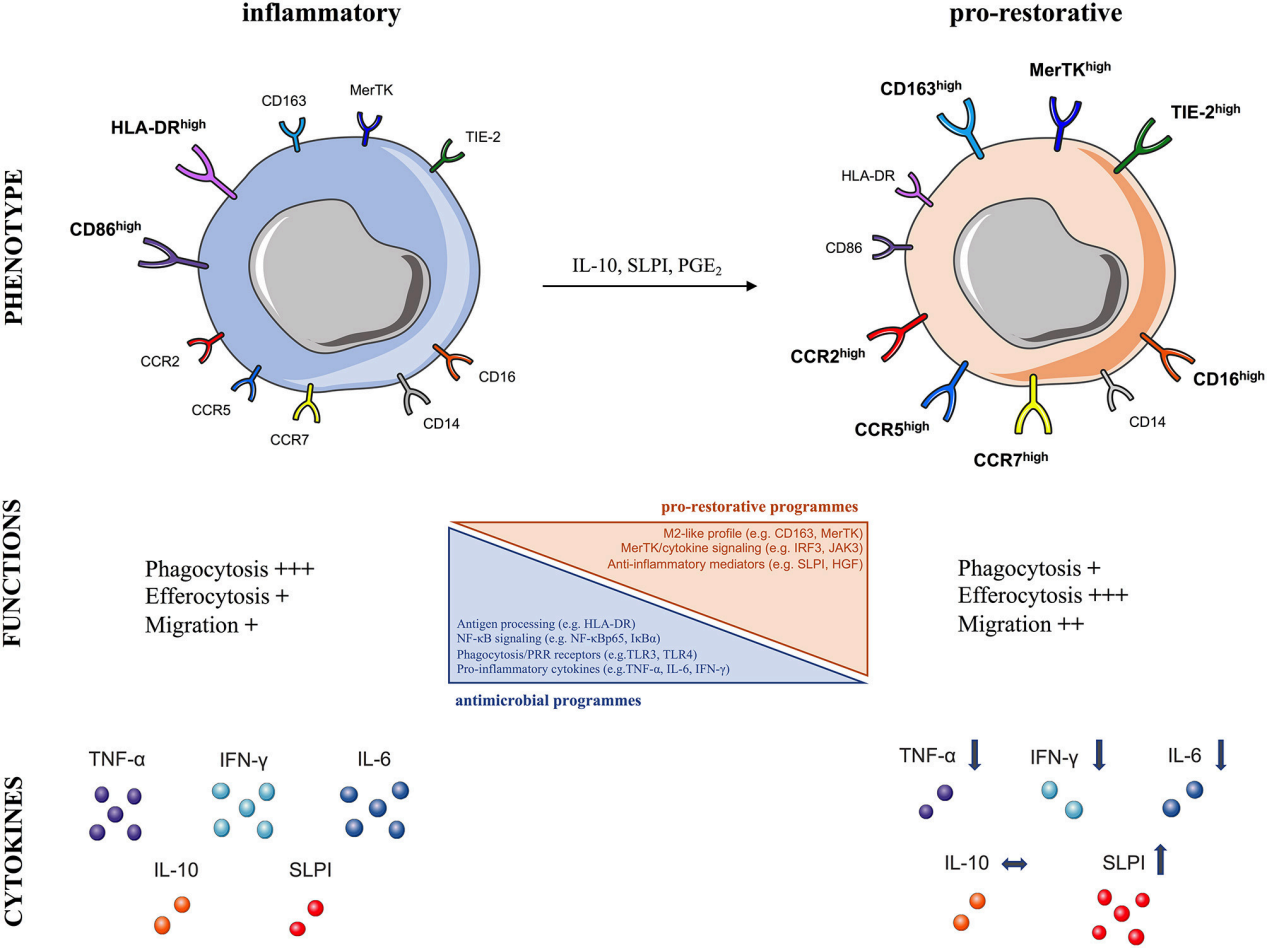
Characteristics of human pro-restorative monocytes and macrophages in acute liver failure syndromes. The schematic summarizes the phenotypic and functional characteristics of **(Left)** steady-state inflammatory and **(Right)** pro-restorative monocytes and macrophages described in acute liver failure syndromes, which can arise in response to micro-environmental cues (IL-10, SLPI, PGE_2_), CCR, CC-chemokine receptor; CD, cluster of differentiation; HGF, hepatocyte growth factor; HLA-DR, human leukocyte antigen-DR; IFN-γ, interferon gamma; IL, interleukin; MerTK, Mer Tyrosine Kinase receptor; NF-κB, nuclear factor-κB; PGE2, prostaglandin E2; SLPI, secretory leukocyte protease inhibitor; Tie2, angiopoietin receptor: TNF-α, tumor necrosis factor-alpha; TLR, Toll-like receptor.

In line with these notions, we have provided evidence of peripheral immunosuppression in patients with ALF, also termed “immuneparesis,” showing major functional defects in both innate and adaptive arms of immunity (64, 77, 102, 103). A hallmark of ALF is that monocytes are functionally impaired and hyporesponsive to microbial challenge (64, 77, 102). Detailed phenotypic characterization of circulating monocytes *ex vivo* in ALF patients revealed an immunosuppressive phenotype, typified by reduced HLA-DR expression but increased CD163, Tie-2, and MerTK expression (64, 66, 77) (Figure 3). Additionally, ALF monocytes show increased expression of tissue (CCR2, CCR5) and lymph-node (CCR7) homing receptors (64, 66, 77). Functional analyses show that ALF monocytes produce less pro-inflammatory (e.g., IFN-γ, TNF-a, IL-6) cytokines after microbial challenge, through NF-kB pathway inhibition, but have enhanced anti-inflammatory (e.g., SLPI) mediator secretion (64, 66, 77, 104) (Figure 3). Furthermore, monocytes in ALF patients have impaired *E. coli* bacteria uptake and enhanced efferocytosis of apoptotic cells (64, 66, 77, 104).

In support of these findings, we showed that MerTK expression by monocytes/macrophages indicates pro-restorative yet immunosuppressive functions in liver failure syndromes (66, 77). MerTK, a tyrosine-kinase receptor mainly expressed by monocytes/macrophages, is a negative regulator of pro-inflammatory TLR signaling pathway (43). We have shown that MerTK+ monocytes in ALF secrete reduced pro-inflammatory cytokines after microbial challenge (66, 77). Furthermore, transcriptional analyses of MerTK+ monocytes in ALF revealed marked reductions in a number of immune-regulatory pathways, including antigen-processing (e.g., HLA-DRA), TLR and NF-κB signaling (e.g., NFKBIA, NFKBIZ, TLR4), phagocytosis/PRR receptors (e.g., FCGR2A/C, FCGR3A/B), and cytokines (e.g., TNF) (66). These cells have an M2-like skewed profile (e.g., CD163), active MerTK/cytokine down-stream signaling (e.g., IRF3, JAK3) with concomitant down-regulation in cell activation related genes (e.g., NLRP3) (66) (Figure 3). These transcriptomic profile of monocytes is consistent with the impaired monocyte antimicrobial responses (cytokines, phagocytosis) described *ex vivo* in ALF (64, 66, 77, 104). In addition, MerTK+ monocytes possess enhanced trans-endothelial migratory characteristics which enable them to accumulate in diverse sites of inflammation, so they are found in the liver, lymph nodes, and circulation of patients with ALF (66, 77).

The systemic and hepatic micro-environmental milieu is a critical determinant of monocyte and liver macrophage function during ALF. For instance, *in vitro* incubation of healthy monocytes in the presence of ALF patient derived plasma or liver homogenates induces similar to *ex vivo* monocyte characteristics, described above (55, 64, 66). This work suggests that anti-inflammatory/regenerative mediators (e.g., IL-10, SLPI) that are produced in the inflamed liver, and which serve to dampen pro-inflammatory responses and limit the extent of injury, are of sufficient magnitude to spill-over into the systemic circulation where they reprogram circulating monocytes into an immunosuppressive state (55, 64). This functional impairment renders monocytes less able to respond to secondary infectious stimuli, hence compromises host anti-microbial defense mechanisms and may account for the increased susceptibility to infections and poor outcomes from sepsis encountered in ALF patients (63, 64, 102, 105). It is therefore of great importance to understand in more detail the underlying mechanisms of monocyte suppression in ALF, so that novel immune-based therapies can be developed (55, 106).

#### Systemic Immunosuppression in ACLF

Immune dysfunction is central to the pathogenesis of ACLF and is postulated to account for its infectious complications and their negative impact on patient survival (28, 77, 107). Immune dysfunction in ACLF is multifactorial. From a pathophysiological perspective, ACLF is a dynamic, multisystem process that involves several defects/abnormalities in cellular and soluble components of the immune system (108, 109). These defects eventually lead to a state of acquired immunodeficiency (108, 109), impairing the host's antimicrobial responses and thus conferring an increased susceptibility to infections (110, 111). Cellular components involve functionally reprogrammed innate and adaptive immune cells; soluble components include albumin, cytokines, coagulation factors, and the complement system (77, 104, 108, 109). Limitations in the available rodent models of ACLF mean most results are obtained from clinical studies in liver failure patients (10). ACLF's complexity is highlighted by various impairments in different tissue compartments; circulation, gut, peritoneum, liver, and the reticuloendothelial (RES) system, as reviewed with more details elsewhere (108, 109). For instance, increased bacterial translocation of gut-derived organisms to the portal and lymphatic circulations is observed in CLD and ACLF patients, as a consequence of diverse changes; e.g., altered gut microbiota composition and increased intestinal permeability (112). This phenomenon is of pathological significance in ACLF, given that gut-derived PAMPs can perpetually stimulate the immune system (112). Furthermore, pathogen clearance mediated by the RES is reduced proportionally to liver dysfunction severity while reduced hepatic synthesis of innate antimicrobial proteins, such as albumin and complement, thus contributing to decreased bactericidal capacity of phagocytic cells (108, 109).

Inflammatory responses in ACLF are also imbalanced, ranging from initial SIRS activation to subsequent CARS development (77, 104, 107–109). ACLF patients are characterized by upregulated circulating levels of both pro-inflammatory and anti-inflammatory cytokines (11, 28, 77). SIRS and CARS are believed to exert crucial modulatory effects on immune cell effector function in ACLF, such as monocytes/macrophages, resulting in defective immune responses to microbial cues (28, 107). This may explain the dynamic immunosuppressive state differences between patients with stable cirrhosis, acute decompensation and ACLF (77, 104, 108, 109). Work from our group has well-described the monocyte and macrophage dysfunction in ACLF patients (77, 104, 111, 113). The main defects include reduced pro-inflammatory cytokine responses to microbial challenge and impaired antigen-presentation capabilities due to reduced HLA-DR expression (77, 111, 113, 114). These attenuated innate responses are considered a state of refractoriness due to recurrent PAMP exposure and physiological adaptation to counter-regulate inflammatory responses, however this favors development of secondary infections and is associated with increased mortality (77, 108).

In a clinical study led by O'Brien et al. the immunosuppressive lipid mediator prostaglandin E2 (PGE2), that inhibits TLR4 protein expression, was detected at increased levels in plasma derived from patients with ACLF (115). PGE2 was shown to inhibit macrophage pro-inflammatory cytokines in response to LPS and decrease macrophage bacterial killing (115). We have revealed another mechanism explaining immuneparesis in ACLF, that involves the activation of MerTK on monocytes/macrophages in the circulation and tissue sites of inflammation in these patients (77). MerTK overexpression conferred a decreased *ex vivo* response to LPS and closely correlated to levels of immunosuppression, SIRS activation, and disease severity scores in ACLF (77). This study proposed MerTK antagonism as a therapeutic strategy to restore innate responses at later stages of ACLF where prolonged CARS predisposes to infectious complications (77). In the same context, we recently demonstrated an expansion of mononuclear CD14^+^HLA-DR^−^ myeloid-derived suppressor cells (M-MDSCs) in the systemic circulation of ACLF patients (104). M-MDSCs in ACLF are highly immunosuppressive: they decrease T cell proliferation, produce less TNF-α following TLR stimulation and have a reduced *E. coli* bacteria phagocytosis (104). M-MDSCs are of great pathological significance in ACLF; given they impair both innate and adaptive responses to microbial agents, they can contribute to the increased frequency of infections encountered in these patients (104). Interestingly, patients with ACLF have a very high short-term occurrence of bacterial and fungal infections (9, 10). Together, these findings suggest that monocyte/macrophage immunosuppression in ACLF develops in parallel to SIRS and this could explain the high risk of nosocomial infections encountered in ACLF patients (77, 108). It is also hypothesized that this immunosuppression might be a regulatory mechanism to limit the monocyte and macrophage responses to elevated amounts of various stimuli (PAMPs, DAMPs, soluble proteins, cytokines and chemokines) (104, 116, 117). However, there is currently no evidence to support this, and future studies are needed to investigate whether the development of systemic inflammation and immunosuppression are related in ACLF (10).

## Immunotherapeutic Strategies to Treat Liver Failure

### Targeting Liver Macrophages: Opportunities and Challenges

Liver macrophages are central to the pathogenesis of ALF and ACLF driving the initiation, propagation, and resolution of injury and related inflammation. Therefore, they are an attractive target for developing new therapeutic approaches to treat such conditions. First, central pathways regulating their recruitment (chemokines), responses to injurious, or infectious insults (e.g., PRRs, DAMPs, PAMPs, inflammasome activation) and differentiation (effector cytokines, polarization markers) are well-conserved between mice and humans, thus allowing translation from animal experimental models to human diseases (2). Second, a number of drugs with good safety profile approved for liver disease patients (e.g., albumin, glucocorticoids, N-acetylcysteine), including those with ALF/ACLF, are shown to exert great immune-modulatory effects on macrophages (108, 118). Third, the high scavenging capacity of liver macrophages allows their preferential targeting using drug carrier materials (hard-shell microbubbles, liposomes, and polymers) (119). Finally, macrophage-related biomarkers may allow patient selection with favored positive responses (55, 75); increased circulating levels of soluble forms of scavenger receptor CD163 (sCD163) and mannose receptor (sMR) detected in ALF/ACLF patients are proposed as surrogate markers of macrophage activation and predict mortality (120, 121).

However, the development and application of macrophage-directed therapies to treat liver diseases may face some challenges (2). First, animal models may exhibit quite opposing functions of macrophage subsets depending on the various applied experimental conditions (55, 75). Thus, for any interventions the optimal dosing, timing, subset-specific targeting in relation to the disease stage must be considered. Second, animal models are not fully representative of the mechanistic spectrum of human diseases but reflect only certain aspects of their pathogenesis. For instance, they develop much more rapidly than human diseases which may affect the macrophage adaptations in response to injury. Mice also bear few immunological differences; for example, the murine liver is largely enriched in NKT cells (122). Furthermore, there is greater heterogeneity in patients compared to inbred mouse strains with respect to intrinsic (genetics, sex, age) or extrinsic (microbiota, infections, medications) factors that might influence macrophages. Third, macrophage heterogeneity and their context-specific functions are currently better understood in the mouse rather than the human liver (1). Currently, there is limited access to liver tissue at the various stages of diseases, while technical difficulties in isolating different macrophage populations hamper the detailed characterization of liver macrophage subsets in humans. Despite these challenges, our detailed understanding of the pathways involved in initial liver injury recognition by resident KCs, amplification of responses by monocyte recruitment to the liver and their context-specific differentiation into diverse liver macrophage subsets have offered exciting platforms for developing novel macrophage-directed immunotherapeutic strategies.

### Inhibition of Kupffer Cell Activation

At the early phases of ALF and ACLF, immune therapies could be targeted at restricting the profound innate immune activation. The initial recognition of liver injury, that is mainly mediated by resident KCs, triggers inflammatory cascades whose activation can be modulated by several approaches (Figure 2B). For instance, the early communication of cellular distress or death from hepatocytes with KCs is through DAMP/PAMP interactions with PRRs and NF-κB signaling and NLRP3 inflammasome activation, so these can be clear targets for immunotherapy. Inhibition of TLR2, TLR3, and TLR4 have been shown to ameliorate liver injury in murine models of APAP hepatotoxicity (19, 123, 124). Furthermore, *in vivo* inhibition of the purinergic receptor P2X7, which acts upstream of the inflammasome and is activated by ATP, has revealed protective effects against APAP injury (38). Another strategy would be to target released DAMPs such as HMGB-1 and histones. Interestingly, HMGB-1 neutralizing antibodies are shown to ameliorate liver injury and reduce bacterial translocation in murine models of paracetamol-induced ALF (21, 32). Further evidence from a rat model of ACLF suggests that blocking HMGB-1 reduces hepatic apoptosis, hepatic inflammatory response and SIRS, thus alleviating inflammation and SIRS in ACLF (125). Hence, pharmacological HMGB-1 blockade in the early stage of human ALF (initiation phase) or ACLF (AD phase) might prevent organ failure so translation of HMGB-1 inhibitors to clinical trials is a potential therapeutic approach (108). Another DAMP, histones, initiate inflammation via TLR2/TLR4 and activation of the inflammasome; animal and human studies showed that targeted anti-histone treatments reduce monocyte pro-inflammatory cytokine production and reduce severity of ALF (36). In the same context, novel drugs targeting inflammasome activation may dampen hepatic pro-inflammatory processes (126).

Increased bacterial translocation, a major contributor to immune dysfunction in ACLF (112), is paralleled by increased gut-derived PAMPs that perpetually stimulate the hepatic innate immune system, magnifying further liver injury (108). Therefore, influencing the gut barrier or the gut microbiome using probiotics or antibiotics could potentially alleviate the pathogenic KC activation (127). Antibiotic agents are widely used drugs for treatment of the infectious complications in ACLF patients (108) while an ongoing study from our group is evaluating the non-absorptive antibiotic Rifaximin that may beneficially influence the gut microbiota to decrease pathologic bacterial translocation. Such therapeutic strategies aimed to limit initial innate immune activation would be most effective if administered very early in disease onset, while levels of DAMPs/PAMPs are high and prior to the propagation phase of liver injury (55). However, this is challenging from a clinical perspective, as the time window for these therapies to be effective is likely to be short. Furthermore, a degree of immune activation is required for resolution of injury, as this initial step leads to recruitment of monocytes and expansion of macrophages at sites of injury, that is associated with effective efferocytosis, pro-restorative functions and facilitation of tissue regeneration (55).

### Inhibition of Monocyte Recruitment to the Liver

Inflammatory monocytes amplify and perpetuate inflammation in liver diseases. Their recruitment to the liver is driven by chemokine-chemokine receptor interactions in animal models and patients, with the CCL2–CCR2 and CCL5/CCR5 as the most prominent pathways in ALF and ACLF (53, 57–59, 77). Many available pharmacological strategies can interfere with this signaling including inhibition of chemokines with small molecule inhibitors, monoclonal antibodies against chemokines or receptors and receptor antagonists preventing chemokine binding (128) (Figure 2B). For example, the CCR2/CCR5 inhibitor cenicriviroc (CVC) has been recently tested in a randomized, double-blind, phase 2b study including a large cohort of patients with non-alcoholic steatohepatitis (NASH) and liver fibrosis (129). CVC was previously found to block monocyte recruitment to the liver and exert anti-fibrotic effects in experimental models of liver and kidney fibrosis (130). This trial revealed that after 1 year CVC treatment, twice as many subjects achieved improvement in fibrosis and no worsening of steatohepatitis, compared with the placebo (129). CVC might also bear therapeutic potential in ALF or ACLF; Mossanen et al. recently showed that the early pharmacological inhibition of either chemokine CCL2 (by the inhibitor, mNOX-E36) or CCR2 (by the CCR2/CCR5 inhibitor, CVC) reduced monocyte infiltration and indices of liver injury in murine APAP-induced ALF (53). Importantly, neither the early nor the continuous inhibition of CCR2 hindered repair processes during resolution from injury. Various other CCR2, CCL2, or CCR2/CCR5 inhibitors are currently developed and tested in other inflammatory/metabolic diseases (e.g., type 2 diabetes), clinical trials are needed though to define their efficacy in liver diseases (128). One important finding from the NASH trial was the excellent safety profile of CVC, supporting that inhibiting inflammatory monocytes might not affect essential macrophage immune responses and antimicrobial defense, in a chronic liver disease at least (129).

### Promotion of Macrophage Polarization and Differentiation

Macrophages exert key functions in liver injury and therefore there is a theoretical concern that targeting them or inhibiting their recruitment could prove counterproductive. Rather, a therapeutic approach utilizing key mediators to promote an early switch in macrophage function to favor a pro-restorative phenotype and thereby accelerate resolution of injury and hepatocyte regeneration should be considered (55) (Figure 2B). Steroids are well-known to promote macrophage anti-inflammatory/resolution responses and therefore their administration might be beneficial in the early phases of liver failure (118). A retrospective analysis of ALF patients did not show an overall survival benefit while transplant-free survival was slightly higher in the steroid-treated group. However, the overall numbers of treated patients in this study were low and steroids were administrated before the onset of ALF in half of the cohort (131). Thus, steroid efficacy in ALF, and their use in ACLF patients (108), is still under debate. As the timing of their administration appears crucial, a prospective trial is suggested to evaluate the steroid effects in the early pro-inflammatory phase of ACLF, so as to limit the initial hepatic inflammation and subsequent SIRS. Steroid usage in the later phases of ACLF, where anti-inflammation prevails, is likely to be detrimental as it may increase predisposition to infection (108).

In contrast to chemokine strategies that reduce monocyte/macrophage recruitment to the liver, there are alternative therapeutic strategies that intentionally cause the opposite, namely augment the macrophage numbers and functions (75) (Figure 2B). Immune dysfunction is common in liver failure syndromes, rendering the patients susceptible to infectious complications. Thus, the effects of hematopoietic growth factors so as to restore immune functions are currently being investigated (132). For instance, granulocyte colony-stimulating factor (G-CSF), produced by macrophages and other immune cells, is shown to reduce disease severity scores, septic episodes and increase survival in a study using G-CSF supplementary for 28 days in the treatment of patients with ACLF (133). This effect may be mediated through mobilization of CD34+ hematopoietic progenitor cells into the liver (133). Furthermore, G-CSF has been shown to improve impaired phagocytosis and bacterial killing by neutrophils *in vitro* and *in vivo* in patients with ALF (134, 135). However, its effects on macrophages are not described. In a similar fashion, the role and therapeutic potential of CSF1 was recently examined in ALF (67). In patients with acetaminophen-induced ALF, low serum CSF1 levels correlated with increased mortality. Exogenous administration of CSF1, in the form of a crystallisable fragment (Fc), promoted hepatic macrophage accumulation in mice (67). CSF1-Fc increased the proliferation of liver-resident KCs and the recruitment of monocytes, promoting their differentiation, which was associated with indicators of increased innate immunity in mice after partial hepatectomy or APAP-induced injury with resident KCs as the main effector cells (67). Thus, patients with impaired macrophage functions such as ALF and ACLF, might benefit from such approach.

Finally, the high scavenging capacity of liver macrophages, especially that of resident KCs, allows their preferential targeting using drug carrier materials such as hard-shell microbubbles, liposomes, and polymers (119). Upon systemic administration in mice, 15–50% of these three prototypic drug delivery systems can be found in the liver, and KCs are the main cellular target for such carriers (119). If particles are functionally endowed with the sugar moiety mannose, the targeting specificity for KCs that carry the mannose scavenging receptor (CD206) can be significantly increased (119). In a proof-of-concept study involving systemic *S. aureus* infection in mice, the liposomal administration of the antibiotic vancomycin efficiently targeted KCs and aided the elimination of a reservoir of *S. aureus* residing within KCs (84). Similarly, the macrophage-targeted delivery of dexamethasone has been shown to reduce fibrosis in mice (136). Taken together, such immunotherapeutic strategies ideally would be able to target liver macrophages without causing a concomitant immunosuppressive effect on circulating monocytes and should allow monocyte-derived macrophage recruitment to sites of tissue inflammation or infection (55). Future studies in mice and humans will provide a better understanding of monocyte and macrophage plasticity in the liver and will hopefully lead to the development of novel therapeutic targets for use in ALF, ACLF, and other inflammatory conditions.

The apparently contradictory aims of some of the described macrophage-targeted approaches highlights the challenges in developing immunotherapeutic strategies for ALF and ACLF. There is an inherent tension between the local hepatic environment, in which promotion of anti-inflammatory programmes is desirable to resolve inflammation and restore tissue integrity, and the systemic immune environment in which enhanced anti-microbial immunity is required. Cellular and humoral components of the response to liver injury, such as MerTK± macrophages and SLPI, which are important in local tissue repair have also been shown to be key drivers of systemic immunosuppression (64, 66, 77, 106). While current experimental tools and models are unable to resolve this paradox, it is hoped that with greater understanding of the inflammation-resolution pathways and the mechanisms of peripheral immuneparesis, macrophage and monocyte targeted approaches could be developed.

## Conclusion

The last two decades have seen a great progression in our understanding of acute liver failure syndromes as inflammatory, immune-mediated conditions. The recognition of ACLF as a condition distinct from decompensated chronic liver disease has enhanced interest, research and understanding. In both ALF and ACLF, monocytes and macrophages play a key role in disease pathogenesis; driving local inflammation, tissue repair, and systemic complications. As research reveals greater levels of complexity and diversity of myeloid cell function in acute liver failure syndromes, both the challenges and opportunities of developing targeted immunotherapy are brought into focus.

## Author Contributions

ET, CA, and LP planned the manuscript. ET and LP wrote the manuscript. ET, KW, MM, and LP reviewed and edited the manuscript.

### Conflict of Interest Statement

The authors declare that the research was conducted in the absence of any commercial or financial relationships that could be construed as a potential conflict of interest.
